# Establishment of an *in vitro* culture model of theca cells from hierarchical follicles in ducks

**DOI:** 10.1042/BSR20160491

**Published:** 2017-05-11

**Authors:** Xiang Gan, Da Chen, Yan Deng, Jusong Yuan, Bo Kang, Jiamin Qiu, Wenqiang Sun, Chunchun Han, Jiwei Hu, Liang Li, Jiwen Wang

**Affiliations:** Farm Animal Genetic Resources Exploration and Innovation Key Laboratory of Sichuan Province, Sichuan Agricultural University, ChengDu, SiChuan, China

**Keywords:** characteristics, culture model, duck, theca cell

## Abstract

Theca cells, including theca interna cells and theca externa cells, are vital components of ovarian follicles. The aim of the present study is to identify a reliable method for the *in vitro* culture of theca cells from duck ovarian hierarchical (F4-F2) follicles. We improved the method for cell separation by using trypsin to further remove granular cells, and we increased the concentration of fetal bovine serum used in *in vitro* culture to improve cytoactivity. Cell antibody immunofluorescence (IF) showed that all inoculated cells could be stained by the CYP17A1/19A1 antibody but not by the FSHR antibody, which could stain granulosa cells. Furthermore, morphological differences were observed between the outlines of theca interna and externa cells and in their nuclei. Growth curve and *CYP17A1/19A1* mRNA relative expression analyses suggested that the growth profile of theca interna cells may have been significantly different from that of theca externa cells *in vitro*. Theca interna cells experienced the logarithmic phase on d1–d2, the plateau phase on d2–d3, and the senescence phase after d3, while theca externa cells experienced the logarithmic phase on d1–d3, the plateau phase on d3–d5, and the senescence phase after d5. Taken together, these results suggested that we have successfully established a reliable theca cell culture model and further defined theca cell characteristics *in vitro*.

## Introduction

Theca cells, including theca externa cells and theca interna cells, are vital components of ovarian follicles, and they originate from fibroblast-like stromal cells in the ovary [1]. As a cell marker of follicle development, the appearance of theca cells is a major marker of the formation of secondary follicles, and this morphological change is highly consistent with the process of follicular development [2,3]. Otherwise, in terms of physiological functions, theca cells interact with granulosa cells and oocytes through members of the autocrine BMP and TGF-β families and other growth factors. They co-regulate follicular recruitment, development, selection, and degeneration [4–6]. Studies have shown that granulosa cells are unable to synthesize steroid hormones in prehierarchical follicles, while almost all steroid hormones are synthesized in theca cells. Even in hierarchical follicles, theca cells are involved in the development and apoptosis of follicles by synthesizing androgen and estrogen [2,7,8]. These facts indicate that theca cells play a key role in the recruitment, development, selection, and apoptosis of avian follicles.

Because of the essential functions of theca cells during the development of avian ovarian follicles, establishing an *in vitro* culture model of theca cells is important and necessary for future investigations. Early in 1973, researchers had begun to preliminarily explore the isolation and culture of the follicular granulosa layer and the theca layer of hens [9–11]. In addition, in 1989, turkey granulosa cells and theca cells were isolated and cultured by Porter et al. [7,12], but all the studies on these cells did not measure or guarantee their viability and purity, nor did they define their characteristics. After these studies, most investigations of the granulosa layer and theca layer of follicles consistently used the previous methods, with no obvious improvements in separation or culture [3,8,13,14]. In other words, the previous studies on avian theca cells did not reliably measure their viability and purity, and their characteristics are not fully understood. However, previous studies proved that the FSHR protein was present only in granulosa cells within follicles, while CYP17A1 and CYP19A1 were present only in theca cells. In addition, assessing the CYP17A1/19A1 content was the best standard for evaluating the synthesis ability of androgen and estrogen in theca externa and interna cells respectively [2,3,8,13,15–20]. The previous studies defined the basic characteristic differences between the granulosa layer and the theca layer and provided the theoretical criteria for identifying the granulosa layer and the theca layer at the tissue level; however, no studies have systematically measured the purity, viability, and characterization of theca cells in birds. A reliable model for avian theca cell culture has not yet been established.

Therefore, in the present study, we improved the methods of theca cell isolation and culture *in vitro*. Specific theca cell proteins were measured and identified using immunofluorescence (IF), and cell viability was evaluated by MTT assay. In addition, the expression patterns of two marker genes (*CYP17A1/19A1*) were also determined by quantitative real-time PCR (qPCR) during theca cell culture *in vitro*. The present study aims to establish a reliable duck theca cell culture model *in vitro* and to further define its characteristics, which might provide a foundation for future studies involving the recruitment, development, selection, and apoptosis of avian follicles.

## Materials and methods

### Animals

Laying Liancheng White ducks (2 years old) were used in the present study. The ducks were kept under natural light and temperature conditions at the Waterfowl Breeding Experimental Farm at Sichuan Agricultural University (Sichuan, China) and were provided unlimited access to food and water. Individual laying cycles were recorded for each duck, and all ducks in the same laying cycle were killed by cervical dislocation 18–20 h after oviposition.

### Isolation and culture of duck theca cells

Follicles from each ovary were separated and subsequently washed in ice-cold sterile phosphate buffered saline (PBS, pH 7.4), and hierarchical follicles (F4-F2) were selected. Tweezers were used to peel away the connective tissue, and then an approximate 2.0–2.5 cm slit was cut with a surgical blade across from the stalk. The yolk and the granulosa layer flowed out. In addition, residual follicular tissues were inverted and washed several times with PBS to wash away the granulosa layer and yolk. The residual follicular tissues were incubated with 0.25% trypsin/EDTA (1×; Gibco) while shaking in a water bath for 10 min to remove the residual granulosa cells and other impurities [7,9,14]. Media (DMEM and F-12/1:1; (HyClone), 10% fetal bovine serum (Gibco), 100 μg/ml streptomycin, and 100 μg/ml penicillin (Gibco)) were added to end the digestion. In addition, the residual follicle tissue was rinsed with ice-cold PBS several times to obtain the clean theca layer. Then, the theca layer was finely minced using scissors and incubated in digestion buffer (PBS, 0.3% collagenase type I (Gibco), 0.1% DNase (Coolaber), 4% BSA (Gibco)) at 37°C while shaking in a water bath for 20 min. The digestion was terminated by the addition of ice-cold PBS. The theca cell suspension was filtered with a 200-mesh filter and then centrifuged at 800×***g*** for 10 min at room temperature to separate floating impurities. The theca cells were cultured in a humidified atmosphere at 5% CO_2_ and 95% air at 37°C. To remove blood cells that could not adhere to the culture plate, the medium was changed after 6 h of incubation. Granulosa cells taken from the same follicles were cultured according to the method reported by Wen et al. [21].

### Dynamic observation and growth of theca cells

Theca cells were seeded on 96-well plates, and their viability was measured every day for 7 days; then, the MTT assay was performed as previously described [22,23]. To each well in the 96-well plate that contained theca cells, 200 μl of 0.5 mg/ml MTT (Amresco) was added (MTT was added in two additional blank wells as controls), and the cells were incubated at 37°C for 4 h. The MTT solution was removed, and then 150 μl of DMSO (Solarbio) was added to each well and the plate was shaken for 10 min. Then, the absorbance at 490 nm was measured by an automatic enzyme immunoassay analyzer [24]. Before measuring the cell viability, the characteristics of duck theca cells were captured using a microscope (Olympus, Tokyo, Japan).

### Cell antibody immunofluorescence

Specific follicle proteins were detected by IF assay. The theca cells were fixed with 4% paraformaldehyde (Solarbio), penetrated with 0.1% Triton-X (Amresco), sealed with 4% BSA (Solarbio), and then washed with PBS. The antibodies (anti-CYP17 rabbit polyclonal (Bioss), anti-CYP19 rabbit polyclonal (Bioss), anti-FSHR rabbit polyclonal (Boster)) were diluted 1:50 and added to the thecal cells (CYP17, CYP19, and FSHR) and the granulosa cells (FSHR) respectively, while the control group was washed in PBS without primary antibodies. All of the groups were incubated at 4°C for 8 h. Next, FITC-goat anti-rabbit IgG (EARTHOX) was diluted 1:200 and added to all the previously mentioned wells at 37°C for 1 h. Then, 1 μl/ml DAPI (Solarbio) was added to the wells for 10 min. Finally, the images were collected by fluorescence microscopy.

### Quantitative real-time PCR

Total RNA was extracted from the cultured cells using TRIzol (Invitrogen) at various time points (d1, d2, d3, d4, d5, d6, and d7) that corresponded to the MTT assay and image collection. First-strand cDNA was synthesized from 10 μg of total RNA using a cDNA synthesis kit following the manufacturer’s instructions (TaKaRa, Shiga, Japan). Levels of *CYP17A1/19A1* were detected using the SYBR PrimerScript^TM^ real-time PCR kit (TaKaRa) and a CFX96^TM^ Real-Time system (Bio-Rad, CA, U.S.A.). The PCR was performed in a 25 μl reaction volume that consisted of 2.0 μl of cDNA, 12.5 μl of SYBR Premix EX Taq, 8.5 μl of sterile water, and 1.0 μl of each gene-specific primer. The raw results were repeated three times and normalized to *β-Actin* and *GADPH* using the 2^−ΔΔ*C*t^ method [25]. Primers for these genes are listed in Table 1.

**Table 1:** Primers used for qPCR

Genes	Sequence (5′–3′)	Temperature (°C)	Size (bp)
CYP17A1	F: GCTCCCTCTGCTTCAACTCCT	60	100
	R: CCTGACCTTGAGGCACTTCTTC		
CYP19A1	F: CTGGTCCTGGTCTCGTGCGTAT	60	139
	R: GATGTGTCAAGCATGATCCGTCTC		
GAPDH	F: AAGGCTGAGAATGGGAAAC	54	254
	R: TTCAGGGACTTGTCATACTTC		
β-Actin	F: GCTATGTCGCCCTGGATTTC	60	168
	R: CACAGGACTCCATACCCAAGAA		

## Results

### Morphological characteristics and growth analysis of theca cells

**Figure 1:**
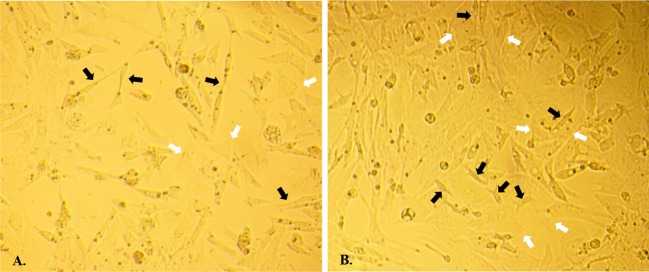
Morphological characteristics of theca cells Morphological characteristics of duck theca cells *in vitro* as seen under a microscope (×200). (**A**) Theca cells *in vitro* cultured at d2. (**B**) Theca cells *in vitro* cultured at d4. The theca interna cells are marked by white arrows, and the theca externa cells are marked by black arrows.

**Figure 2:**
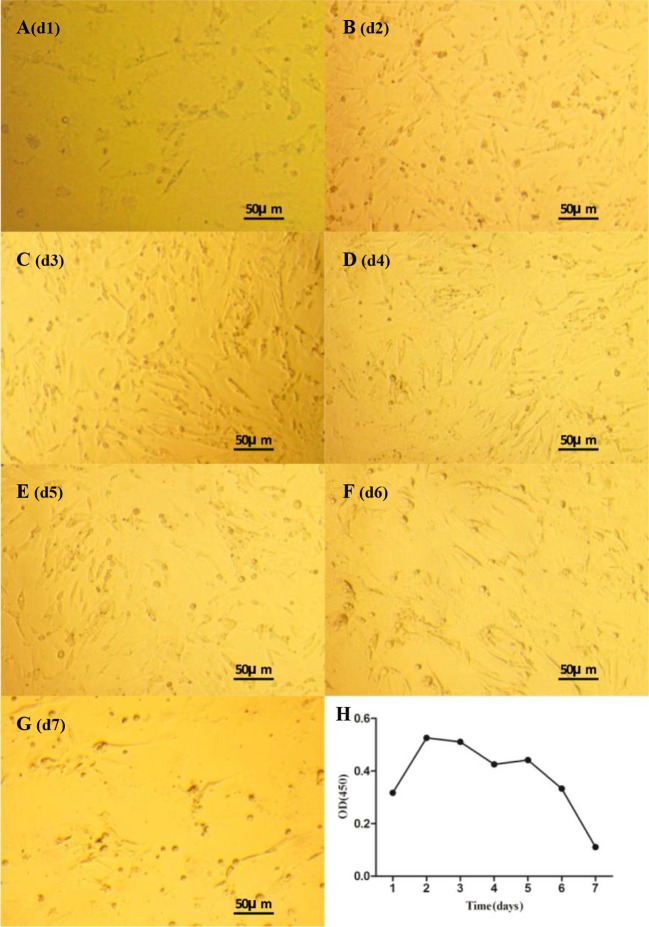
Morphological characteristics and growth curve of theca cells Morphological characteristics and growth curve of duck theca cells *in vitro* as seen under a microscope (×100). (**A**–**G**) Morphology of duck theca cells on d1-d7 *in vitro*. (**H**) Growth curve of duck theca cells that were cultured *in vitro* (*P*<0.05).

Morphological characteristics of duck theca cells cultured *in vitro* recorded on different days are shown in Figure 2. Theca interna cells showed an epithelial-like shape, while theca externa cells were observed to have a strip-like shape (Figure 1). After 24 h of incubation, all theca cells were tightly adhered to the bottom of the plate, and the adhered cell layer was extremely thin. The growth phase lasted until d3, and during this period, theca cells grew very quickly and spread over the bottom of the plate (Figure 2C). At d5, the theca cell density began to decline, and growth declined sharply from d6 to d7 (Figure 2E–G). In addition, it is noteworthy that theca externa cells and theca interna cells did not proliferate independently but grew mutually together.

The theca cell growth curve is shown in Figure 2H. Theca cells grew quickly in the first 2 days, and their viability value peaked at d2. After that, the cells showed two steady trends on d2–d3 and d4–d5 and two declining trends on d3–d4 and d6–d7.

### Expression profiles of *CYP17A1/19A1* mRNA during theca cell culture

**Figure 3:**
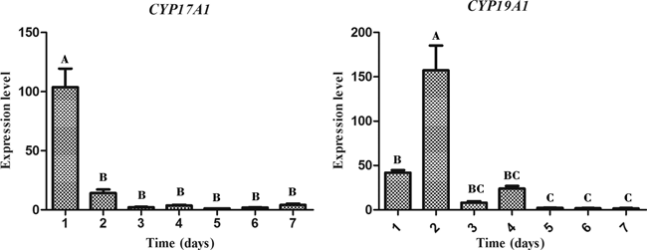
Expression profiles of *CYP17A1/19A1* mRNA during theca cell culture Relative mRNA expression of the *CYP17A1* and *CYP19A1* genes in duck theca cells on d1–d7. The letters at the top of each bar represents the significant differences between the gene expression in various stages (*P*<0.05).

During the culture process (7 days), we measured the mRNA expression profiles of *CYP17A1/19A1* in theca cells each day by qPCR (Figure 3). The level of *CYP17A1* mRNA expression in theca cells on d1 was significantly higher than on the other days. After d1, the expression level of *CYP17A1* decreased sharply. The expression of *CYP19A1* mRNA was high on d1 and d2. On d1, the level of *CYP19A1* mRNA expression was significantly higher than on d5–d7, and it reached its peak on d2. Expression on d2 was significantly higher than on the other days.

### CYP17A1/19A1 and FSHR antibodies used for immunofluorescence identification

**Figure 4:**
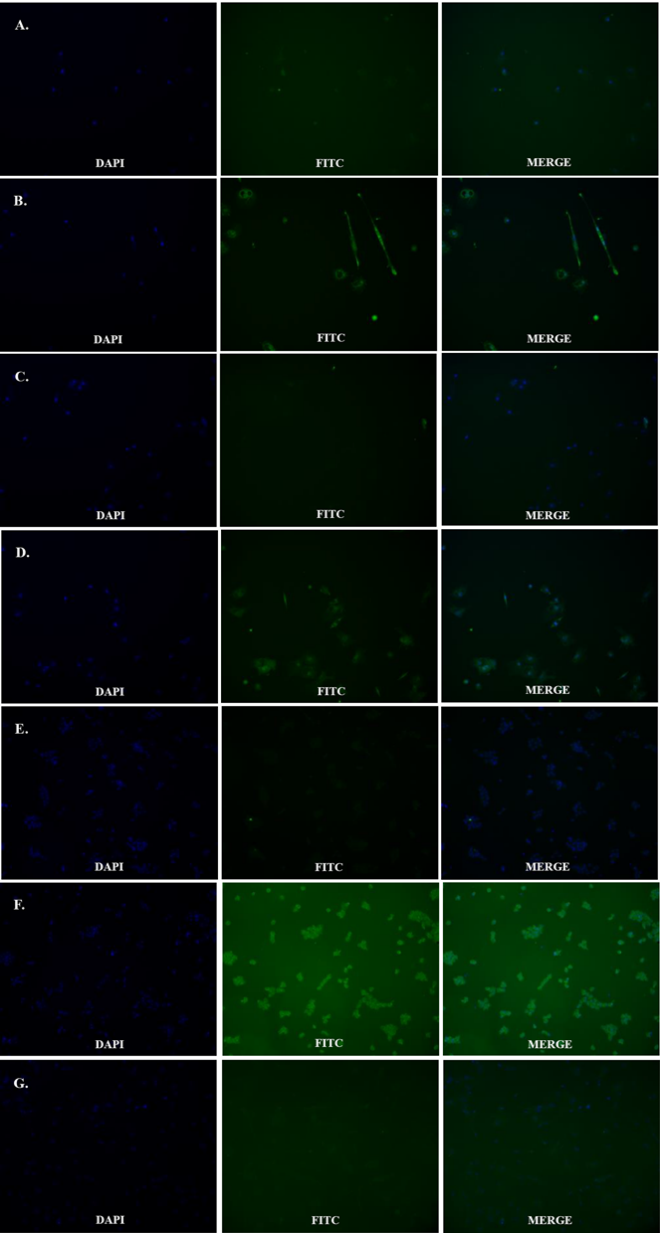
immunofluorescence identification by CYP17A1/19A1 and FSHR antibodies Fluorescent image of theca cells and granulosa cells marked by their specific protein antibodies. (**A** and **B**) Control and treatment groups stained with CYP17A1 antibody to mark theca cells. (**C** and **D**) Control and treatment groups stained with CYP19A1 antibody to mark theca cells. (**E**) Treatment group stained with FSHR antibody to mark theca cells. (**F** and **G**) Control and treatment group stained with FSHR antibody to mark granulosa cells.

CYP17A1, CYP19A1, and FSHR antibodies were used to identify theca cells and granulosa cells respectively. Theca cells reacted with both the CYP17 and 19A1 antibodies and were marked with a green outline (Figure 4). However, when both the theca cells and the granulosa cells reacted with the FSHR antibody, only the granulosa cells were marked with a green outline (Figure 4). In contrast, there were significant differences between the cellular nuclei of theca interna cells and theca externa cells, which were identified by DAPI staining. The nuclei of theca interna cells were observed to be larger and rounder than those of theca externa cells.

## Discussion

Theca cells are important for the recruitment, development, selection, and apoptosis of avian follicles. Early in 1979, researchers had explored methods for separating avian theca cells. In hens, Huang et al. [9] used a simple physical method to directly separate theca cells. To date, this method is still widely used in avian species [14,26–28], but it might be difficult to separate theca cells from granulosa cells completely *in vitro* using this method. Previous reports [26,27] and our unpublished results suggested that the sensitivity of granulosa cells to trypsin was more intense than theca cells. Therefore, to ensure that the residual granulosa cells adhered to the theca layer were completely removed, trypsin was used to further digest theca cells after their physical separation. In previous studies, theca cell digestion methods were not stable in mammals or birds, and both the digestion reagent and the digestion time were not clearly defined [9,12,14,26–34]. Previous theca cell culture experiments in birds were limited to turkey and chicken species, and all these experiments only used collagenase as the digestion reagent. The digestion time was vague and ranged from 35 to 120 min [9,12,14,26–28]. In this experiment, we found that if only collagenase was used to digest theca cells, it produced a large amount of goo, which had serious impact on the ability to separate and collect the cells. Therefore, in the present study, a mixture of collagenase and DNase was employed to digest the cells and the digestion time was adjusted to 20 min. These modifications achieved satisfactory results.

In mammals, we commonly authenticate the purity of theca cells by a radioimmunoassay (RIA), because various follicular cells have obvious differences in their hormonal content [30,33–35]. However, in birds, the various components of follicular cells (including granulosa cells, theca interna cells, and theca externa cells) contain similar hormones (although the content varies greatly) [7,8]. Therefore, we cannot use RIA to authenticate avian theca cells. It has been reported that in hen follicular cells, the CYP17A1/19A1 protein exists specifically in theca cells (including theca interna cells and theca externa cells) on an organizational level [8,13,15,18]. Therefore, we used the CYP17A1/19A1 antibody to authenticate duck theca cells using the IF technique. These results showed that all theca cells that were separated and cultured in the present study were stained with the CYP17A1/19A1 antibody (Figure 4A–D). It verified that all cells that were isolated in the experiment were theca cells. In addition, we further used the FSHR (the specific avian granulosa cells protein) [19] antibody (Figure 4E and F) to ensure that there were no granulosa cells mixed in with the theca cells. Based on these results, we corroborate that the methods for isolation and digestion used in the present study are reasonable and reliable.

During the experiment, we found that the two types of cells had distinct morphological characteristics. The shape of theca externa cells appeared similar to smooth muscle cells because of their strip-like shape, while theca interna cells looked like typical steroidogenic cells because of their epithelial-like shape. The nuclei of theca interna cells appeared larger and rounder than theca externa cells (Figure 1), and all these results were consistent with previous studies [36–39]. Furthermore, the cell growth trend in the present study was evaluated by the MTT assay, and it was determined to be compliant with basic cell growth laws [40,41]. The viability of theca cells also remained high, which indicated that theca cells could maintain normal morphology and good activity in this cell culture model. The fact that the expression levels of *CYP17A1/19A1* mRNA reached certain threshold values could reflect the steroid synthesis ability and viability of theca cells [3,18]. In our study, the expression of *CYP17A1* mRNA was significantly higher on d1 (*P*<0.05) than at other time points, which suggested that *in vitro*, androgen was synthesized in copious amounts from theca interna cells at the beginning of the culture. On the second day, the expression of *CYP19A1* mRNA was significantly higher (*P*<0.05) than at the other time points, and when combined with the high quantity of androgen synthesized on the first day, the results suggested that most of the androgen in theca externa cells was transformed into estrogen by the CYP19A1 protein on the second day. These steroid synthesis patterns and characteristics are consistent with previous reports [2,3,16,18,20], which indicate that the ability of theca cells to synthesize steroids in this culture model remained normal. After comprehensive analysis of theca cell density, viability, and assessment of their ability to synthesize steroids during d1–d7, we found that all parameters of cell density, viability, and expression of the* CYP17A1/19A1* genes did not have a steady trend after seeding, which suggests that the latency period of theca cells should be between d0 and d1. The rapid growth period occurred between d1 and d2, which revealed that the logarithmic phase of theca cell growth should be between d1 and d2. After d2, there were two distinct trends, which could correspond to the two different cells. Combined with the qPCR results, we presumed that the growth plateau phase of theca interna cells was from d2 to d3, and on approximately the third day, cell activity began to decrease. For theca externa cells, their plateau phase could have been around d3–d5, with cell activity beginning to decrease on d5. However, in our research, the trends of theca cell density, viability, and their ability to synthesize steroids were mutually consistent. Further investigations need to be performed to clarify the details of the growth characteristics of theca interna and externa cells.

In conclusion, the present study summarized and improved upon the previous methods for theca cell separation and culture, established a reliable duck theca cell culture model *in vitro*, and further defined the morphology, growth trends, and basic characteristics of theca cells.

## Abbreviations

BMP: bone morphogenetic protein
CYP17A1: cytochrome P450 17α-hydroxylase
CYP19A1: cytochrome P450 aromatase
DMEM: dulbecco's modified eagle media
DMSO: dimethyl sulphoxide
FSHR: follicle simulating hormone receptor
GAPDH: glyceraldehyde-3-phosphate dehydrogenase
IF: immunofluorescence
MTT: 3-(4,5-dimethylthiazol-2-yl)-2,5-diphenyltetrazolium bromide
qPCR: quantitative real-time PCR
RIA: radioimmunoassay
TGF: transforming growth factor
